# Supplementary material for: Deconstructing celebratory acts following goal scoring among elite professional football players
Source: PLoS One. 2020 Sep 16;15(9):e0238702. doi: 10.1371/journal.pone.0238702 (PMC7494092; doi:10.1371/journal.pone.0238702)
Supplement: Attachment — Submitted filename: Letter for reviewers (1).docx [file pone.0238702.s002.docx]

May 2, 2020

Dear Prof. Christian Stamov Roßnagel
Academic Editor
PLOS ONE

We would like to thank you as acting editor and the distinguished reviewers for making prodigious effort in reviewing the manuscript and overall providing positive feedback for the article. Your comments were invaluable and allowed us to considerably improve the manuscript. We appreciate your time and efforts in advancing the article. We did our best to accommodate *all* of your recommendations.

We hereby attaching, as requested, 3 files consisting of:

a. Rebuttal letter that responds to each point raised by the academic editor and reviewer(s). We have uploaded this as separate file and labeled 'Response to Reviewers'.
b. A marked-up copy of our manuscript that highlights changes made to the original version. This file was uploaded as separate file and labeled 'Revised Manuscript with Track Changes'.
c. An unmarked version of our revised paper without tracked changes. This file was uploaded as separate file and labeled 'Manuscript'.

Following is a listing of reviewers’ comments and our responses to the comments.

| **Reviewer 1- Comments** | **Responses to Comments** |
| --- | --- |
| We note that Figures 2, 3, 4 includes an image of a patient / participant / in the study. If you are unable to obtain consent from the subject of the photograph, you will need to remove the figure and any other textual identifying information or case descriptions for this individual. | We changed the pictures into caricatures/cartoons which do not require any copy right and consent – |
| Please ensure that all your conclusions are sufficiently supported by data and/or references. For example, you have stated that:  “However, in the context of maintaining their culture, during times of celebration, players from Africa and South America demonstrate performances of religion far more than their European counterparts. This is given the fact that the nonreligious population of Europe is far greater than that of Africa or South America (see World Economic Forum website, 2019).” But have not proven this link in the context of your research; thus, this should be presented as a hypothesis not as an statement. | We thank the reviewer for her/his important comment, and in the revised manuscript, we added:  This is given the fact that the nonreligious population of Europe is far greater than that of Africa or South America (see World Economic Forum website, 2019). After all, sports cannot be disentangled from larger social practices such as religion and politics. In both Africa and South America, sport is intertwined with control of the body and space and has become closely associated with social identity and expression of a people's social character (Vidacs, 2006; Arbena, 2012). For example, in Africa, sports serve to teach moral values such as religion, and "the maintenance and transformation of identities can be examined beautifully through sports" (Vidacs, 2006, p. 337). |
| Thank you for including your ethics statement:  "It was approved by the University’s ethical committee and sent only to the PLOS ONE journal." Please amend your current ethics statement to include the full name of the ethics committee/institutional review board(s) that approved your specific study. | Done when uploading |
| Once you have amended this/these statement(s) in the Methods section of the manuscript, please add the same text to the “Ethics Statement” field of the submission form (via “Edit Submission”). | Done when uploading |
| Please include your tables as part of your main manuscript and remove the individual files. Please note that supplementary tables (should remain/ be uploaded) as separate "supporting information" files | Done when uploading |
| The [PLOS Data policy](https://urldefense.com/v3/__http:/www.plosone.org/static/policies.action*sharing__;Iw!!PhOWcWs!gyPbCiVdYnk5sYIIasGK0eYJyDpt5EmDs8CZL9hxraEbQxDvfq5nhPJoLUGMGV4Sf8PMVA$) requires authors to make all data underlying the findings described in their manuscript fully available without restriction, with rare exception (please refer to the Data Availability Statement in the manuscript PDF file). The data should be provided as part of the manuscript or its supporting information, or deposited to a public repository. For example, in addition to summary statistics, the data points behind means, medians and variance measures should be available. If there are restrictions on publicly sharing data—e.g. participant privacy or use of data from a third party—those must be specified. | Data Attached |
| Major comments: The introduction could be more focused. Please make sure to be explicit about the rationale of the paper and the aim(s) of this research. Also, if you do decide to conduct Null-Hypothesis-Significance-Testing, make sure to report hypotheses based on sound theory. | We thank the reviewer for this comment. In the revised paper, Goffman's framework has been refined to be more focused and more clearly address the rationale of the research:  Goffman's (1969) dramaturgical perspective is considered a critical cultural analysis (Birrell & Donnelly, 2004; Lev, 2020), and provides a solid conceptual framework to players' goal celebrations. According to Goffman, social interaction, such as goal celebration, is a stage where the player strives to promote his/her honest and unbiased social identity. In this reality, the performer is a "fabricator of impressions involved in the all-too-human task of staging a performance" (Goffman, 1969, p. 252). Specifically, the player is an actor who has *Front* and *Back regions of behavior*. 'Front region' refers to the place the performance is given, where the actor engages in and performs his/her role for the audience while the 'Back region of behavior' is considered a place where the performer can step out of character and cease to 'play a part' for an audience. Moreover, according to Goffman, to construct and maintain a trustworthy and credible impression as part of the Front region, one must be persistent in conveying expression in both the 'gives' and 'gives-off' channels. The 'gives' channel involves, for the most part, the *verbal symbols* used and conveyed purposefully, while the 'gives-off' channel involves a range of various actions transmitted via *nonverbal symbols* such as bodily gestures and props. This paper centers on the players' celebrations ('gives-off') in front of the fans and cameras (i.e., "front region of behavior"). Detecting the players' performances on the football field via Goffman's theoretical perspective is useful given the fact that: On the dramaturgical dimension, sport competition is defined as a play/spectacle, all actors acting towards putting the competition on stage in front of the public. The focus is on the performance act and on the way sport actors manage to respond to the social constraints and expectations attached to their role (Dumitriu, 2014, p. 508). There is no sound theory to explain celebratory actions in soccer/football. We have established a completely new innovative conceptual framework which consists of related concepts from dynamical systems, social emotions, and Gofman’s front and back stage However, we tried to focus the aims of the study and give it an exploratory flavor. Thus, no hypotheses have been postulated, and the inferential statistics do not relate to working hypotheses but rather generalization of findings. |
| An important issue in this paper regards sampling celebratory behavior in soccer. Where all matches of an entire season of the Champions League analyzed? More information on this is needed to convince readers that there was no selection bias in the study and that the sample studied is representative considering the claims the authors would like to make. | We sampled all 366 goals scored at the Champions League (exclusive of qualification sequences) from 125 games played. There was no selection criteria; thus, no selection bias: Every UEFA Champions League begins with a double (round-robin) group stage of 32 teams, which since the 2009–10 season is preceded by two qualification sequences for teams (N=79 at the 2018/9 season) that do not receive direct entry to the tournament. |
| The most important problem with this paper at present is the coding of the celebratory behavior. This is not a trivial issue as coding whole body movements in non-standardized real-life contexts that were filmed for different durations and from different perspectives is highly challenging. Hence, it is insufficient to state that one person was trained to code the body language. There is a vast literature base on behavioral coding in psychology. But the authors do not refer to any of this literature and I am skeptical if the method used in this research is reliable. The authors need to substantially elaborate on this. This information is essential for me to make an informed recommendation concerning publication of this research. | We appreciate this important comment. We have used a rigorous procedure for coding nonverbal behaviors and added this into the article as following:  According to Dael, Mortillaro, and Scherer (2012) there are several methods of coding body movement in nonverbal behavior research, but they claim there is no consensus on a reliable coding system that can be used for the study of emotion expression. They proposed a new integrative method, which they termed “the body action and posture coding system.” Of the three dimensions they proposed (anatomical, form, and function), we relied mainly on observing the form of the goal celebration acts but assigned their functions to individual and social roles driven from sound theoretical foundation, which led to a newly established model which guided our study.  Video recordings of the celebration acts were transcribed verbatim immediately after each observation by one of the authors who holds expertise in body expression. The coder acquired a vast formal education and experience in teaching, coding, and interpreting body motions of artists, politicians, and instructors. To finalize the coding scheme, a negotiated approach was taken wherein the main coder and the other authors discussed actively the identified codes, while aiming to form a final version with the entire coded celebratory actions (Garrison, Cleveland-Innes, Koole, & Kappelman, 2006). The discussions resulted in a final full agreement about the coded behaviors. All the coded behaviors expressed positive emotions of joy, pride, and happiness. However, the coding system did not allow for distinguishing among the designated emotions, but rather distinguished among single, double, and team modes of goal celebration.  To ensure quality and trustworthiness, we followed Tracy's (2010) conceptualization of quality. First, our study focused on a new topic with meaningful implications for society given the significant role soccer plays in society world-wide. It also holds significant value for understanding acts which express joy, pride and happiness which have both personal and social roles. A unique model was developed to represent these expressions of emotions in the form of body language. The data obtained herein clarify the relationship between the celebratory action and social facilitation of these acts. Additionally, to gain a meaningful coherence within our data, the stated study's objectives were followed, and the selected exploratory research design and data analysis procedures aligned with these objectives. We have established a solid theoretical model which was associated with the study's objectives, methodology, and findings. In the process of data collection, appropriate behavioral coding practices were conducted using full agreement among the main coder and his affiliates. |
| A related point. The authors seem to have missed a lot of relevant literature in setting up this research, especially on nonverbal behavior coding and nonverbal behavior in sports. | We have added the following: In sport, nonverbal behaviors were studied only recently (Bijlstra, Furley & Nieuwenhuys 2020; Brimmell, et al., 2018; Furley, Dicks & Memmert 2012; Furley, Moll, & Memmert, 2015; Furley & Schweizer (2014a; 2014b; 2016); Greenlees, Bradley, Holder, & Thelwell, 2005; Moesch et al., 2015; Moll, Jordet, & Pepping, 2010), and for the most part (with the exception of Furley & Schweizer 2014a and Moesch, 2015) not in the context of goal |
| At the end of the introduction you state that you analyzed 125 matches in the European Premier League during 2019. Champions League seems the less confusing description here. | As explained earlier, We sampled all 366 goals scored at the Champions League (exclusive of qualification sequences) from 125 games played. There was no selection criteria; thus, no selection bias: Every UEFA Champions League begins with a double (round-robin) group stage of 32 teams, which since the 2009–10 season is preceded by two qualification sequences for teams (N=79 at the 2018/9 season) that do not receive direct entry to the tournament. |
| It’s Kahneman not Khanman. By the way, dual-process theorizing is quite common in sports and the authors might want to incorporate some of the dual-process work in sports into their manuscript. | Thank you for this observation. We have corrected the spelling error. Kahneman’s 2-systems was incorporated to our innovative model, which guide the research. |
| Is the historical information in the section “the Champions League framework” necessary? This section seems not very important towards the papers aims. | We deleted this section from the article. |
| This is not a very important point, but is it correct that “The UEFA Champions League final is the most watched annual sporting event in the world”? I thought this was the Super Bowl. | Indeed, correct! – in 2019 380 Millions watched the CL final and “only” 102 saw the Superbowl.. |
| I don’t understand how 79 teams (from 54 European associations) can be correct |   79 teams from 54 of the 55 UEFA member associations participate in the 2018–19 UEFA Champions League (the exception being Liechtenstein, which does not organise a domestic league). The association ranking based on the UEFA country coefficients is used to determine the number of participating teams for each association:   - Associations 1–4 each have four teams qualify. - Associations 5–6 each have three teams qualify. - Associations 7–15 each have two teams qualify. - Associations 16–55 (except Liechtenstein) each have one team qualify. |
| While revising your submission, please upload your figure files to the Preflight Analysis and Conversion Engine (PACE) digital diagnostic tool, [https://pacev2.apexcovantage.com/](https://urldefense.com/v3/__https:/pacev2.apexcovantage.com/__;!!PhOWcWs!gyPbCiVdYnk5sYIIasGK0eYJyDpt5EmDs8CZL9hxraEbQxDvfq5nhPJoLUGMGV4w9NQJsQ$). PACE helps ensure that figures meet PLOS requirements. To use PACE, you must first register as a user. Registration is free. Then, login and navigate to the UPLOAD tab, where you will find detailed instructions on how to use the tool. If you encounter any issues or have any questions when using PACE, please email us at [figures@plos.org](mailto:figures@plos.org). Please note that Supporting Information files do not need this step. | Done |
| Style | We have altered the style to Vancouver Style |

Hope you will find this version worthy of publication,

Assaf Lev, Yair Galily, Omer Eldadi & Gershon Tenenbaum
