# Supplementary material for: Deconstructing celebratory acts following goal scoring among elite professional football players
Source: PLoS One. 2020 Sep 16;15(9):e0238702. doi: 10.1371/journal.pone.0238702 (PMC7494092; doi:10.1371/journal.pone.0238702)
Supplement: Attachment — Submitted filename: Response to Reviewers.docx [file pone.0238702.s003.docx]

August 17, 2020

Dear Prof. Christian Stamov Roßnagel
Academic Editor
PLOS ONE

We would like to thank you as acting editor and the distinguished reviewers. We appreciate your time and efforts in advancing the article. We did our best to accommodate the minor revisions requested.

We are hereby attaching, as requested, 4 files consisting of:

a. Rebuttal letter that responds to each point raised by the academic editor and reviewer(s).
b. A marked-up copy of our manuscript that highlights changes made to the original version. This file was uploaded as a separate file and labeled 'Revised Manuscript with Track Changes'.
c. An unmarked version of our revised paper without tracked changes. This file was uploaded as a separate file and labeled 'Manuscript'.
d. A revised data document. This file was uploaded as a separate file and labeled, ‘Data’.
e. A revised figures document in color. This file was uploaded as a separate file and labeled, ‘Figures’.

Following is a listing of reviewers’ comments and our responses to the comments.

| **Reviewer 1- Comments** | **Responses to Comments** |
| --- | --- |
| As one minor revision, to avoid a potential misunderstanding, please acknowledge in the manuscript that a limitation of the study was that it utilized an ad-hoc coding procedure and we do not know how reliable the coding was. | We thank the reviewer for her/his important comment, and in the revised manuscript, on page 11, we added: Nevertheless, it should be acknowledged that a limitation of the study was that it utilized an ad-hoc coding procedure and there may not be consensus as to its reliability. |
| On p. 10 you write "According to Dael, Mortillaro, and Scherer (2012) ... there is no consensus on a reliable coding system", which may be taken to imply that intercoder reliability may not be determined; however, Dael and colleagues did just that in the quoted paper. | We appreciate the insightful comment and in the revised manuscript, we refer to Dael, Mortillaro, and Scherer’s work on intercoder reliability on pages 10-11. |
| The second minor change concerns the original data that should be in English. Please change this. | Changes have been made in the data document. |

Hope you will find this version worthy of publication,

Assaf Lev, Yair Galily, Omer Eldadi & Gershon Tenenbaum
